# Wiring surface loss of a superconducting transmon qubit

**DOI:** 10.1038/s41598-024-57248-y

**Published:** 2024-03-27

**Authors:** Nikita S. Smirnov, Elizaveta A. Krivko, Anastasiya A. Solovyova, Anton I. Ivanov, Ilya A. Rodionov

**Affiliations:** 1 FMN Laboratory, Bauman Moscow State Technical University, Moscow, Russia 105005; 2 Dukhov Automatics Research Institute, VNIIA, Moscow, Russia 127030

**Keywords:** Quantum information, Superconducting devices

## Abstract

Quantum processors using superconducting qubits suffer from dielectric loss leading to noise and dissipation. Qubits are usually designed as large capacitor pads connected to a non-linear Josephson junction (or SQUID) by a superconducting thin metal wiring. Here, we report on finite-element simulation and experimental results confirming that more than 50% of surface loss in transmon qubits can originate from Josephson junctions wiring and can limit qubit relaxation time. We experimentally extracted dielectric loss tangents of qubit elements and showed that dominant surface loss of wiring can occur for real qubits designs. Finally, we experimentally demonstrate up to 20% improvement in qubit quality factor by wiring design optimization.

## Introduction

Quantum processors and simulators comprising tens or even hundreds superconducting qubits have recently been demonstrated^1–5^. Quantum gates errors hinder further size and complexity growth of superconducting circuits and quantum algorithms. On the one hand, reducing two-qubit gate errors to less than 0.1% opens a practical way to implement quantum error correction codes^6,7^. On the other hand, with a reduced gate errors a useful quantum advantage can be achieved near term using variational quantum algorithms and error mitigation^1,8^. However, superconducting quantum bits have natural internal sources of noise and decoherence limiting quantum gates fidelity.

A large part of qubits loss is due to microscopic tunneling defects, which form parasitic two-level quantum systems (TLS)^9,10^ and resonantly absorb electric energy from the qubit mode dissipating it into phonons or quasiparticle bath^11–14^. It is well-known, that such defects reside in the interfaces and surface native oxides around qubit electrodes: metal-substrate (MS), substrate-air (SA), metal-air (MA)^15–19^. This source of qubit loss could be mitigated by reducing the amounts of lossy dielectrics (minimizing Josephson junction area^20,21^, using better materials and defect-free fabrication techniques^22–24^). Another approach for loss mitigation is increasing qubit footprint^25–27^ by minimizing an electric field in the interfaces and preventing TLS excitation due to coupling with their dipole moment.

Qubit relaxation caused by dielectric losses could be decomposed into participations from each material and qubit components:

1 $$\frac{1}{{T}_{1}}=\frac{\omega }{Q}=\omega \sum_{i}{p}_{i}{{\text{tan}}\delta }_{i}+{\Gamma }_{0}$$

where ${T}_{1}$, $\omega$ and $Q$ are the relaxation time, angular frequency and quality factor of the qubit, ${{\text{tan}}\delta }_{i}$ is the dielectric loss tangent of the *i*th material or component, ${p}_{i}$ is their participation ratio defined as the fraction of electric field energy stored within this material or component, ${\Gamma }_{0}$ is the relaxation rate caused by non-surface losses.

**Figure 1:**
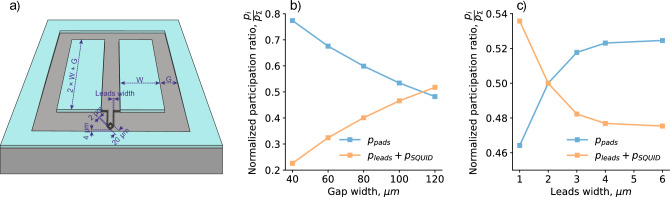
Flux-tunable floating transmon qubit participation ratios. (**a**) Sketch of transmon qubit with a standard wiring and SQUID loop (black) close to control line. Capacitor pad dimensions and gap width (G) are proportional to make qubit symmetrical in both directions. (**b**) Normalized participation ratio of the pads (blue dots) and wiring (orange dots, include leads and SQUID loop) vs. gap width (G). For each gap width capacitor pad width is adjusted to make qubit charge energy ${E}_{C}$ equal to 220 MHz. Leads width is 2.5 µm. (**c**) Normalized participation ratios vs. leads width. Gap width (G) and pad width (W) are chosen equal to 120 µm and 204 µm, correspondingly. Curves flatten out when the participation ratio of the SQUID loop in wiring becomes dominant.

One can imagine a superconducting transmon qubit^28^ as a non-linear LC oscillator, where the Josephson junction or SQUID define a non-linear inductance and the superconducting metal pads define a capacitor. Josephson junction or SQUID loop electrodes have to be electrically connected to the capacitor pads. Such connection is commonly realized as a thin metal wire, which we call ***leads*** in this paper. The design of the frequency-tunable two-padded floating transmon qubit which is investigated in this study is shown in Fig. 1a. Usually, in order to improve qubit relaxation time (dilute an electromagnetic field and lower interfaces participation ratio), the gap (G, Fig. 1a) between the capacitor pads is increased. However, it requires long Josephson junction connecting wires. Moreover, in case of a flux-tunable qubit the wiring length becomes even longer to form a SQUID loop and move it closer to the gap edge and flux-control line. Figure 1b demonstrates qubit pads and qubit wiring (leads and SQUID loop) participation ratios versus qubit gap width. One can notice, as the gap width increases the capacitor pads participation ratio decreases, but the leads and SQUID participation ratios increase. When the gap width is more than 110 µm, then the participation ratio of the leads with SQUID become dominant and further gap widening is impractical. Thus, a relaxation time of a properly designed qubit is limited by the leads and SQUID loss, if their loss tangent is comparable to the capacitor pads one. To further increase the qubit relaxation time, optimization of the lead’s width is required as illustrated in Fig. 1c.

We calculated the participation ratios using a similar method as in Ref.^25^, but with modifications to be able to analyze asymmetrically located SQUID loop with long leads (see supplementary material, Sect. I for details). When we calculate the participation ratios of qubit components, we consider bulk superconductor and crystalline dielectric to be lossless (${tan\delta }_{i}<{10}^{-8}$ for silicon^29^ and sapphire^30,31^), so that:

2 $${{\text{p}}}_{{\text{pads}}}= {{\text{p}}}_{{{\text{pads}}}_{{\text{MA}}}}+{{\text{p}}}_{{{\text{pads}}}_{{\text{SA}}}}+{{\text{p}}}_{{{\text{pads}}}_{{\text{MS}}}}$$

3 $${{\text{p}}}_{{\text{SQUID}}}= {{\text{p}}}_{{{\text{SQUID}}}_{{\text{MA}}}}+{{\text{p}}}_{{{\text{SQUID}}}_{{\text{SA}}}}+{{\text{p}}}_{{{\text{SQUID}}}_{{\text{MS}}}}$$

4 $${{\text{p}}}_{{\text{leads}}}= {{\text{p}}}_{{{\text{leads}}}_{{\text{MA}}}}+{{\text{p}}}_{{{\text{leads}}}_{{\text{SA}}}}+{{\text{p}}}_{{{\text{leads}}}_{{\text{MS}}}}$$

In order to mitigate surface dielectric loss in qubits, previous work was primarily focused on capacitor pads design modifications^25–27,32^, investigation of Josephson junction contribution with so-called bandages^33–35^ and fabrication improvements^33,36–39^. Despite the remarkable results achieved in these works, contribution from the Josephson junction wiring has mainly remained ignored with rare exceptions. In Ref.^25^ there were extracted participation ratios for junction leads, but only for 3D cavity qubits with a single Josephson junction. Recent study^40^ has analytically predicted, that a significant fraction of surface loss comes from the wiring that connects the qubit to the capacitor. In this work, we experimentally studied the contribution of leads and SQUID to the overall qubit surface dielectric loss. We performed finite-element electromagnetic simulations of the transmon qubits with different leads geometries in order to analyze their contribution to the surface losses. Then, we experimentally measured qubits relaxation times and extracted the dielectric loss tangents of the qubit elements associated with the capacitors, leads and SQUID. We also compared two different methods for leads fabrication: etch and lift-off. Then, we demonstrated good agreement between the measured qubit quality factors and the proposed model, so it could be used to further qubit design optimization reducing surface dielectric losses. The dielectric loss model and loss tangents extracted in this study can be applied to improve perspective superconducting qubit, e.g. fluxoniums^41^.

## Results and discussion

**Figure 2:**
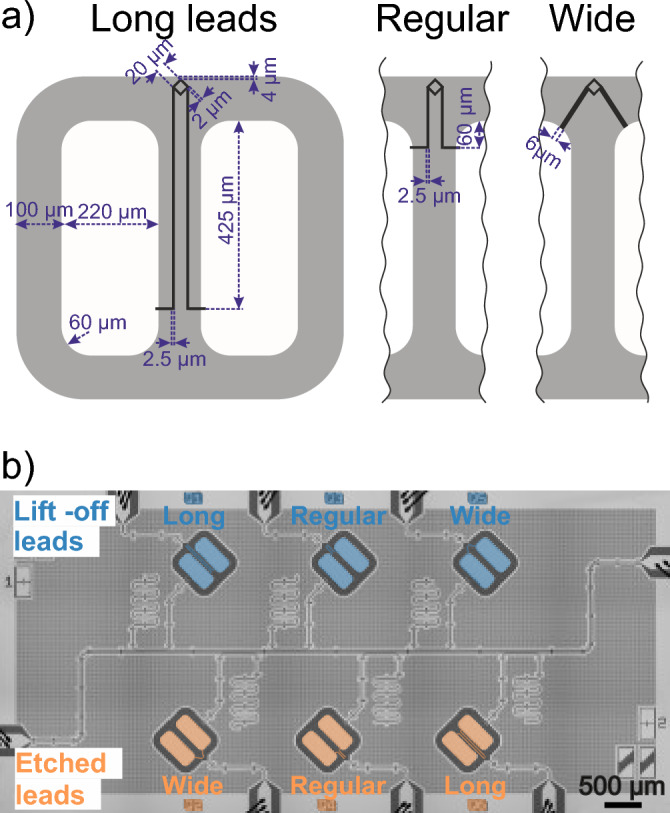
Tunable floating transmon qubit designs and fabricated sample. (**a**) Designs of three transmon qubits with “long leads”, “regular leads” and “wide leads”. Dimensions of square SQUID loop and capacitor pads are fixed for all three designs. Gaps are shown in grey and capacitor pads with the surrounding ground plane in white. (**b**) False-colored SEM image of the sample featuring six qubits coupled to readout resonators. Qubits are initialized and readout is performed via the single feedline. The qubits with etched Josephson junction leads are colored in orange and with lift-off leads in blue.

To analyze the contribution of capacitor pads, leads and SQUID in the total qubit loss, we fabricated six tunable floating transmon qubits on the same chip, so we can assume the same loss tangents of each interface for all the qubits. The fabricated qubits have the same design, except different wiring geometry, as shown in Fig. 2a. They were designed to accentuate participation in the Josephson junction leads. Therefore, we refer to the designs as “long leads”, “regular leads” and “wide leads”. We placed the SQUID loop close to the ground plane to allow for a sufficient inductive coupling with the flux control line. We are able to detune qubits over a wide frequency range. We do not expect the qubits be limited by the dielectric losses in the SQUID loop—ground plane capacitance, since the highest electric field density is concentrated within 2–3 µm of the loop. The calculations of participation ratios were performed considering 3 nm thick dielectric interface layers MA, SA and MS with fixed dielectric constant $\epsilon =10$. Table 1 summarizes the parameters of the fabricated qubits.

**Table 1:** Parameters of fabricated qubits.

Qubit# (lift-off/etch)	Design	${p}_{pads}$	${p}_{leads}$	${p}_{SQUID}$	${f}_{q,max}$, (GHz)	${\eta }_{q}$, (MHz)
1/6	long	1.852	3.312	0.613	4.825/4.739	179
3/4	regular	1.938	1.247	0.694	4.999/5.164	215
5/2	wide	2.086	0.652	0.724	4.824/5.152	182

Qubit numbers correspond to the numbers on Fig. 2b. Etch and lift-off are methods of leads fabrication. Participation ratios of the elements ${p}_{i}$ are multiplied by ${10}^{4}$. ${f}_{q,max}$ and ${\eta }_{q}$ are the experimentally measured qubit frequency at the “sweet spot” and anharmonicity.

In order to estimate the effect of fabrication process to the leads surface losses, we compared two methods of leads patterning. Three qubits on the chip have the leads fabricated together with the SQUID loop using lift-off process as in Ref.^42,43^. The leads of the other three qubits were patterned using optical lithography and then wet-etched together with the capacitor pads. Further details of the fabrication process are provided in the supplementary material, Sect. II. A scanning electron microscope (SEM) image of the sample with six floating transmon qubits is shown in Fig. 2b.

All the qubits are individually coupled to $\lambda /4$ resonators with different frequencies ranging from 6 up to 6.45 GHz for dispersive readout. State-dependent dispersive shifts, qubit-resonator detuning and resonator widths are designed to both distinguish the readout signals and push the Purcell-limited relaxation time as high as possible (> 1 ms), so it does not affect the qubits relaxation time (${T}_{1}$).

**Figure 3:**
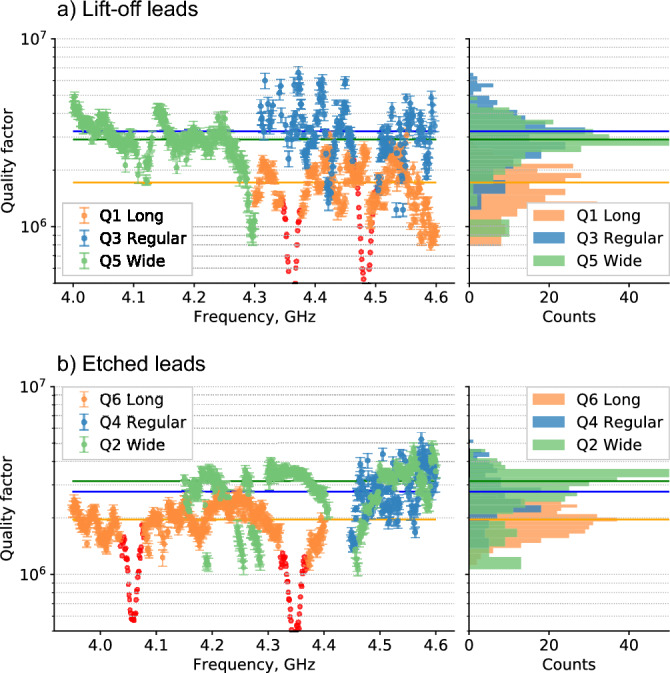
Measured qubit quality factors vs. qubit frequency. Orange, blue and green dots correspond to the “long leads”, “regular leads” and “wide leads”, respectively. Error bars are quality factor fit errors. All the dots with big errors (> 10%) are excluded from the plot. Parasitic modes dots are colored in red and are excluded from the distribution plots. Solid horizontal lines show the median quality factor values. (**a**) Qubits with lift-off leads. Median Q-factors are Q_Q1_ = ${1.78\times 10}^{6}$, Q_Q3_ = ${3.17\times 10}^{6}$, Q_Q5_ = ${2.99\times 10}^{6}$. (**b**) Qubits with etched leads. Median Q-factors are Q_Q6_ = ${1.98\times 10}^{6}$, Q_Q4_ = ${2.76\times 10}^{6}$, Q_Q2_ = ${3.30\times 10}^{6}$.

In this work we measure ${T}_{1}$ of the flux-tunable floating transmons as a function of their frequency. We recalculate ${T}_{1}$ in quality factors (Q-factor) and determine their mean value and confidence interval for each qubit. The experimental pulse sequence we used to measure ${T}_{1}$ at a single frequency is the follows: the qubit is initialized in the $\left|1\right.\rangle$ state by a microwave drive pulse, flux-tuned to the frequency of interest, where we wait for a varied delay time, and then measure the qubit state. To obtain one ${T}_{1}$ curve, we repeat this experiment for 21 equally-spaced delays with 4000 shots each in the range 10 to 400 μs. Using this sequence, we swept the qubit frequency over a range of at least 300 MHz with 1 MHz step. It took us approximately 6 h to measure the entire 300 MHz spectrum. Qubit’s spectra, converted into quality factors, and distributions are presented in Fig. 3. We notice that qubit spectra have Lorentzian-like regions with strong relaxation (red dots in Fig. 3a and in Fig. 3b). We attributed these resonances to the modes of qubit flux control lines or microwave package cavity modes, as their shape maintained after repeated cooldowns. These peaks can be successfully suppressed with IR eccosorb-filters in flux control cables^44^. However, these filters may slightly limit ${T}_{1}$, that is why in this study we don’t use IR filtering for the flux control cables (see the supplementary material Sects. III and IV for more experimental setup details and Lorentzian fitting respectively). We excluded Lorentzian regions from the analysis, as they are not connected with surface dielectric loss.

We compared the qubit components loss tangents extracted with surface loss extraction (SLE)^19^ process (see Table 2). We assumed the loss tangent of pads and SQUID the same for “etched leads” and “lift-off leads” qubits. Qubit quality factor, written through Eq. 1, can be represented in a matrix form as a function of participation matrix $[P]$ (number of columns equal to number of qubit components, including pads, SQUID, lift-off and etched leads; number of rows equal to number of qubits) and loss tangent vector $[{\text{tan}}\delta ]$ (number of columns equal to the number of qubit components plus offset relaxation rate ${\Gamma }_{0}$ assumed constant since qubits were measured in a narrow frequency range):

**Table 2:** Experimentally extracted loss tangents of qubit elements.

Process	${{\text{tan}}\delta }_{pads}$(${\times 10}^{4}$)	${{\text{tan}}\delta }_{leads}$(${\times 10}^{4}$)	${{\text{tan}}\delta }_{SQUID}$(${\times 10}^{4}$)
Lift-off	$11.9\pm 3.1$	$9.4\pm 4.4$	$4.1\pm 1.5$
Wet etch		$7.9\pm 3.7$	

Loss tangents extracted using SLE process for the qubit elements by the leads patterning process. The extracted offset relaxation rate ${\Gamma }_{0}$ is $204\pm 63 {{\text{s}}}^{-1}$.

5 $$\left[1/Q\right]=\left[P\right][{\text{tan}}\delta ]$$

**Figure 4:**
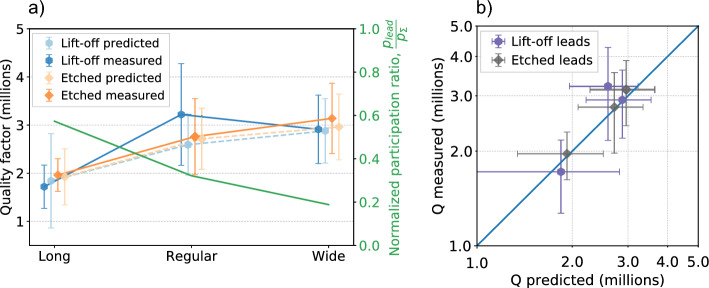
Measured and predicted qubits quality factors. (**a**) Qubits quality factors and normalized participation ratio as a function of leads geometry. Each data point represents median quality factor obtained from measured qubit ${T}_{1}$ spectra (solid lines) or sampled quality factors (dashed lines). Dark blue and dark orange points represent quality factors for measured qubits with lift-off and etched leads, respectively. Light blue and light orange points represent predicted quality factors for lift-off and etched leads, respectively. Plotted normalized participation ratio of leads (solid green line) shows that leads have non-negligible effect. (**b**) Predicted quality factors compared to measured quality factors of qubits with etched (dark gray) and lift-off (purple) leads. The blue line represents perfect agreement between the measured and predicted quality factors. All the error bars on the plots correspond to 68% confidence interval.

In order to determine the uncertainty of the extracted loss tangents, we perform Monte Carlo simulations. We sampled 10,000 Q-factors using mean values and standard deviations taken from the experimental data. Then, taking each sampled Q-factors and calculated participation ratio in the matrix $[P]$, we found the least square solution for the loss tangents using Eq. 6. Using the extracted loss tangents and calculated participation ratios in Eq. 1, we determined the predicted Q-factors, shown in the Fig. 4a as dashed lines. Dark blue dots with solid lines (Fig. 4a) show median values of the measured Q-factors versus leads design for lift-off leads (orange dots with solid lines for etched leads). The horizontal and the vertical errors bars correspond to the standard deviation error of the measured and simulated Q respectively. The goodness-of-fit is shown in Fig. 4b, where we plot the mean measured Q-factors against the mean predicted Q-factors. One can notice quality factors improvement as Josephson junction leads participation ratio decreases. This trend shows that leads with a high participation ratio suppress significantly the qubit quality factor. Qubits with “wide” shortened leads have a higher median quality factor (Q_Q2_ = ${3.30\times 10}^{6}$, Q_Q5_ = ${2.99\times 10}^{6}$) compared to qubits with narrower and longer “regular” leads (Q_Q3_ = ${3.17\times 10}^{6}$, Q_Q4_ = ${2.76\times 10}^{6}$), the worst case is “long” lead (Q_Q1_ = ${1.78\times 10}^{6}$, Q_Q6_ = ${1.98\times 10}^{6}$). The only exception in this experiment is the qubit (Q3) with lift-off “regular” leads, which median quality factor become a slightly higher than for qubit (Q5) with “wide” leads. We assume this to the dynamics of strongly coupled TLS ^45,46^, as the qubit (Q3) has the worst scatter in quality factors data $.$

We attribute the uncertainty of the extracted loss tangents to several sources. First, the scatter in qubit’s quality factor measurement statistics results in wider range of possible linear equations solutions. Second, although we strived to maximize the participation ratio of Josephson junction leads, we were not able to make a design with the participation of the specific component totally prevailing over the other qubit components. The maximum participation ratio of Josephson junction leads relative to the total ratio of all the qubit components in our set of designs was 0.364. The loss tangent of etched leads is smaller than lift-off one. We attribute this to a degraded MS interface due to e-beam resist residuals^47^ and a worse MA interface, which was additionally passivated after shadow deposition (a thicker amorphous oxide can occur). We also note that the cross-section of shadow-evaporated structures is quite complex in practice. There are both exposed oxidized areas of bottom electrode and areas of top electrode metal covering the bottom one. This feature distorts the cross-section of lift-off wiring and SQUID, making them different from a rectangle, which introduces additional MA interfaces. As it is quite complicated, we do not take it into account in our model for participation ratios simulation, that could affect the results accuracy. One can see, that loss tangent of the SQUID loops is lower than the leads for both fabrication routes. The SQUID loops have much smaller footprint area, but still non-negligible TLS defect induced losses.

We simulated qubit spectra by randomly sampling TLS in the qubit interfaces. The simulation shows that only a small fraction of coupled defects is located within the wiring interfaces ($\sim$ 18%), however, their coupling strength is much higher due to the stronger electric fields, which results in non-negligible loss of the wiring elements. We applied the SLE to the simulated data and extracted the loss tangents of the qubit elements similar to the experimentally extracted loss tangents. See the supplementary material, Sect. V for the details.

## Summary

We have studied the wiring surface dielectric loss of superconducting transmon qubits and have found out that relaxation time of qubits with a large footprint is limited by dielectric loss in the leads. We considered three different transmon geometries and experimentally extracted the loss tangents of the capacitive pads, leads and SQUIDs using the SLE process. It is demonstrated, that for a commonly used tunable floating transmon qubit design, which we called "regular" in the work, leads and SQUIDs contribute about 50% of the total dielectric loss. We do not include variation of the capacitor pads participation ratios, which could improve the accuracy of extracted loss tangents, meanwhile, we confirmed, that leads can be a limiting factor for qubit relaxation times.

Wiring leads are often fabricated together with Josephson junctions using lift-off process, which introduce additional surface dielectric loss. We experimentally extracted the loss tangents for wet etched (${tg\delta }_{leads}=(7.9\pm 3.7)\times {10}^{-4}$) and lift-off (${tg\delta }_{leads}=(9.4\pm 4.4)\times {10}^{-4}$) leads. In order to minimize internal qubit surface loss and improve relaxation time, one should fabricate wiring leads together with capacitor pads using etching process. A further leads loss tangent reduction may be achieved by reducing their participation ratio with a substrate trenching^19,29,32,48^.

Finally, we demonstrated that electric field dilution by increasing the wiring width improves qubit performance up to 20% for the considered qubit designs. Further optimization of leads design, for example, by tapering^40^ may increase a relaxation time even more. In addition, one should pay attention to SQUID design, as it has a high participation ratio and loss tangent.

### Supplementary Information

Supplementary Information.

## Data Availability

The data that support the findings of this study are available from the corresponding author upon reasonable request.
