# Supplementary material for: Wiring surface loss of a superconducting transmon qubit
Source: Sci Rep. 2024 Mar 27;14:7326. doi: 10.1038/s41598-024-57248-y (PMC10973359; doi:10.1038/s41598-024-57248-y)
Supplement: Supplementary file 1 — Supplementary Information. [file 41598_2024_57248_MOESM1_ESM.docx]

***Supplementary Material to***

**Wiring surface loss of a superconducting transmon qubit**

**Nikita S. Smirnov,^1,2^ Elizaveta A. Krivko,^1^ Anastasiya A. Solovyova,^1^ Anton I. Ivanov, ^1^ and Ilya A. Rodionov^1,2*^**

^1^FMN Laboratory, Bauman Moscow State Technical University, Moscow, 105005, Russia

^2^Dukhov Automatics Research Institute, VNIIA, Moscow, 127030, Russia

* [irodionov@bmstu.ru](mailto:irodionov@bmstu.ru)

### I. Details on surface participation simulation

The use of participation ratios is convenient for estimating dissipative losses in systems with dielectrics. The method for calculating qubit participation ratios used in the paper is based on the Ref. [1] with some modifications. In this method, calculations are carried out in two steps: a 3D simulation of the entire qubit at a coarse scale (~ μm) (Fig. S1a) and fine simulation of qubit regions with highly concentrated electric fields at the edges of the electrode pads and narrow Josephson junction wiring. Dividing the simulation into stages helps to overcome the problem of obtaining convergent values of electric fields in thin dielectric layers, caused by the large difference between the length scales of the system.

| 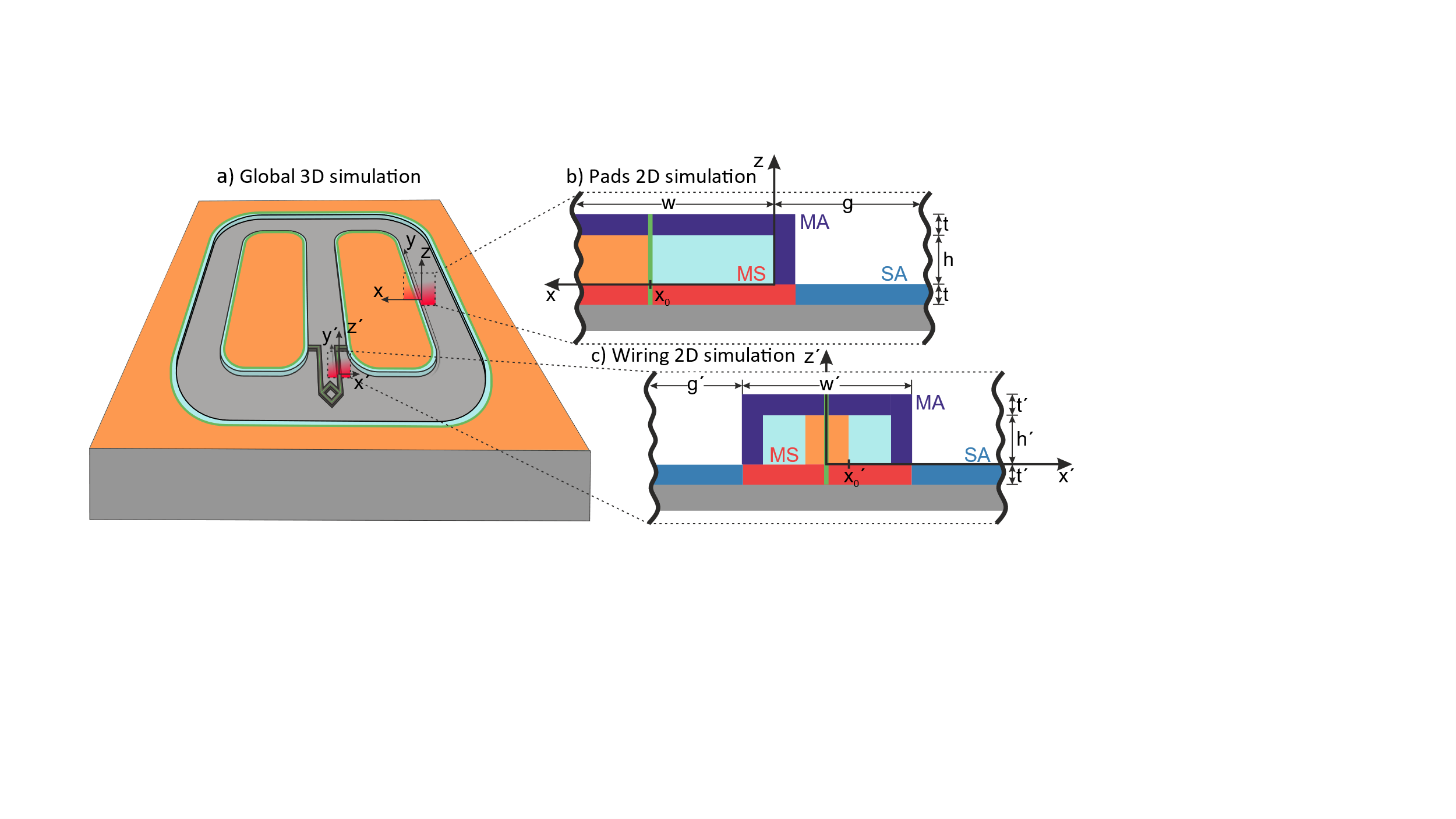 |
| --- |
| **Fig. S1 Illustration of the two-step computation of transmon qubit surface participation ratios.**  **a** The global coarse 3D simulation which includes features of the entire qubit, such as substrate, capacitor pads, ground plane, leads and SQUID loop. Conductors are perfectly-conducting sheets, interface layers are omitted. The inner (orange) and perimeter (blue) regions are separated by the green contour. The red windows represent the placements of the cross-sections. **b** Cross-section of the capacitor pad edge used for 2D electrostatic simulation. Interfaces with thickness of *t* are shown in red (MS), purple (MA) and blue (SA). Superconductor regions with thickness of *h* are represented in orange that is inner region and light blue that is perimeter region. Ground plane edge has the same cross-section. **c** Cross-section of the wiring, used for 2D simulation of wiring, including long leads and a SQUID loop. All color codes are the same as for the cross-section of the capacitor pad edge. |

First, the electrode pads and ground plane are simulated (Fig.S1b). Electrode pads are divided into «perimeter regions» and «inner regions» with a boundary set at a distance $x_{0}$ from the edge equal to 1 $\mu m$*.* Electric field of the inner regions easily converges in 3D simulations since it is located far away from the edge of the electrodes and uniformly distributed. From these regions we immediately extract the electric field values on the top and bottom surfaces of the electrode pads and ground plane, surrounding qubits ($\boldsymbol{E}_{MA}\left( x,y \right)$ and $\boldsymbol{E}_{MS}\left( x,y \right)$), as well as on the surface of exposed substrate areas $\boldsymbol{E}_{SA}\left( x,y \right)$. The field distributions are then used to calculate the inner participation ratios of the regions associated with the capacitor pads, substrate and ground plane:

| $p_{i,int}=t\iint_{int} \frac{\epsilon}{2}\left\vert\boldsymbol{E}_{i}(x,y) \right\vert^{2}dxdy/U_{tot}$ | (S1) |
| --- | --- |

where $i$ is MA, MS or SA, $U_{tot}$ is the total electric field energy in the entire model, $t$ is the thickness of the dielectric layer, which we assumed to be equal to 3 nm in our calculations. Figure S2 illustrates the results of coarse electric field simulation.

| 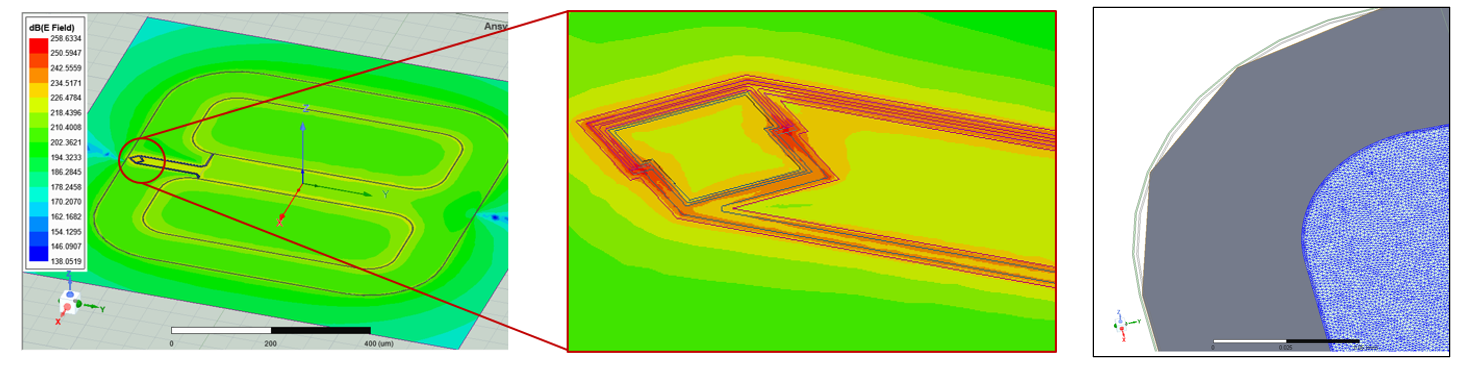 |
| --- |
| **Fig. S2 Resulting electric filed distribution from coarse global simulation and meshed qubit edge.** The electric field is highly concentrated around the Josephson junction wiring. The maximum mesh element length is 1 $\mu m$*.* |

At the second stage, the perimeter regions are calculated. Here, in a 2D simulation, a constant ratio $F$ of the integrated field energy within the entire cross-section of the perimeter region ($0<x<x_{0}$) and the section, that converges both in the coarse 3D simulation, and the 2D simulation $(x_{0}/2<x<x_{0})$. This constant scaling factor is then used to calculate participation ratios of the entire perimeter region:

| $p_{i,per}=F_{i}t\int_{x_{0}/2}^{x_{0}} dx\oint_{y} \frac{\epsilon}{2}\left\vert\boldsymbol{E}_{i}(x,y) \right\vert^{2}dy/U_{tot}$ | (S2) |
| --- | --- |

Electric field distribution in the volume at the edges of the electrode $f(x,z)$ is independent of the pad width, gap width and the other distant boundary conditions so that $E\left( x,y,z \right)=C\left( y \right)f(x,z)$. Figure S3a shows spatial distribution of the electric field $f(x,z)$ for different pad width $w$ and gap width $g$, including typical values, used in this study. Using the simulated $f(x,z)$ scaling factors $F_{i}$ for the electrode pads within 2D cross-section are simulated:

| $F_{MS}=\frac{\int_{0}^{x_{0}} dx\int_{-t}^{0} f^{2}\left( x,z \right)dz}{\int_{x_{0}/2}^{x_{0}} dx\int_{-t}^{0} f^{2}\left( x,z \right)dz}$ | (S3) |
| --- | --- |
| $F_{MA}=\frac{\int_{0}^{x_{0}} dx\int_{h}^{h+t} f^{2}\left( x,z \right)dz+\int_{-t}^{0} dx\int_{0}^{h+t} f^{2}\left( x,z \right)dz}{\int_{x_{0}/2}^{x_{0}} dx\int_{h}^{h+t} f^{2}\left( x,z \right)dz}$ | (S4) |
| $F_{SA}=\frac{\int_{0}^{-x_{0}} dx\int_{-t}^{0} f^{2}\left( x,z \right)dz}{\int_{x_{0}/2}^{x_{0}} dx\int_{-t}^{0} f^{2}\left( x,z \right)dz}$ | (S5) |

Figure S3b shows scaling factor of the MS interface as a function of $x_{0}$ position for different geometries.

| 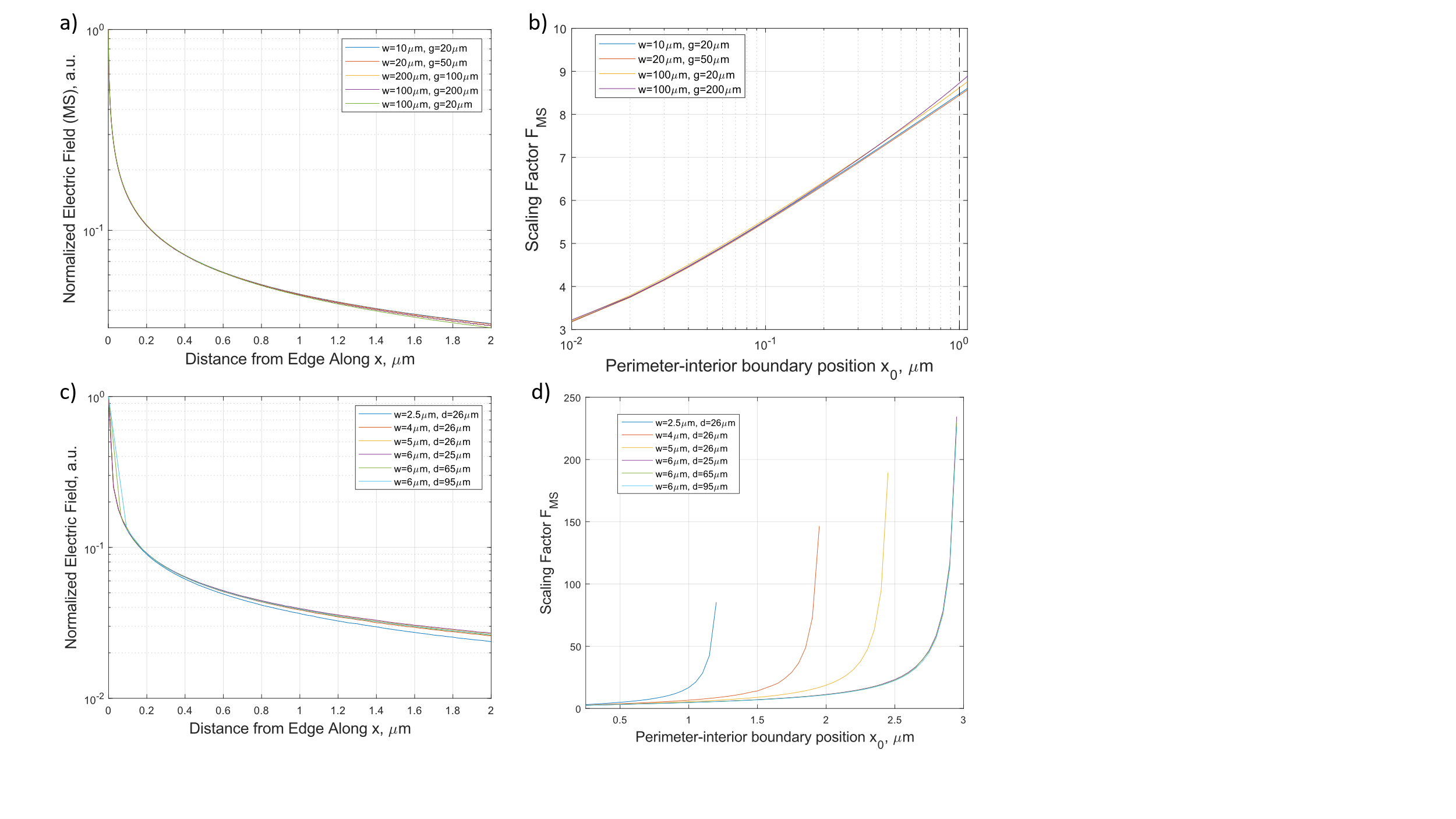 |
| --- |
| **Fig. S3 Results of cross-sectional electric field simulations.** Values are plotted for different distant boundary conditions. *w* represents width of the conducting feature, *g* and *d* represent distance between features for capacitor edge and leads respectively. **a** Normalized electric field distribution within a cross section of the MS interface near a capacitor edge. **b** $F_{MS}$ scaling factors as function of $x_{0}$ position plotted for various boundary conditions. **c** Normalized electric field distribution within a cross section of the SA interface between the wiring features near the lead edge. **d** $F_{MS}$ scaling factors as function of $x_{0}^{'}$ position plotted for various wiring geometries. $F_{MS}$ is the same for all varied boundary conditions when $x_{0}^{'}=0.5 \mu m.$ |

The participation ratios of the qubit wiring are calculated similarly to the capacitor pads (Fig. S3c). Since in our qubit geometry the wiring leads are parallel to each other, we can, as for the capacitor pads, find a constant ratio $F^{'}$ in 2D simulation, and then using the field distribution from the global coarse 3D simulation, calculate the participation ratios. The spatial field distribution at the edges of the leads $f^{'}(x,z)$ is independent of the lead width and distance between the leads for the wiring geometries used in our study. Figure S3c shows normalized field distributions $f^{'}(x,z)$ within SA interface for different leads width $w^{'}$ and distance between the leads $g^{'}$. In 2D simulation we consider scaling factor $F^{'}$ of the entire leads cross-section ($-w^{'}/2<x<w^{'}/2$) and the section, that converges in both coarse global simulation and fine 2D simulation ($-x_{0}^{'}<x<x_{0}^{'}$). The distance $x_{0}^{'}$ within which electric fields converge in both types of simulation is set to be equal 0.5 $\mu m$. For better convergence we set the finer meshing on the leads than on the capacitor pads. Scaling factor is then used to calculate participation ratios of the wiring.

| $p_{i,leads}=F_{i}^{'}t^{'}\int_{{-x}_{0}^{'}}^{x_{0}^{'}} dx^{'}\oint_{y^{'}} \frac{\epsilon}{2}\left\vert\boldsymbol{E}_{i}(x^{'},y^{'}) \right\vert^{2}dy^{'}/U_{tot}$ | (S6) |
| --- | --- |

Scaling factors $F_{i}^{'}$ within the wiring cross-section are calculated as follows:

| $F_{MS}^{'}=\frac{\int_{{-w}^{'}/2}^{w^{'}/2} dx^{'}\int_{-t^{'}}^{0} f^{2}\left( x^{'},z^{'} \right)dz^{'}}{\int_{{-x}_{0}^{'}}^{x_{0}^{'}} dx^{'}\int_{-t^{'}}^{0} f^{2}\left( x^{'},z^{'} \right)dz^{'}}$ | (S7) |
| --- | --- |
| $F_{MA}^{'}=\frac{\int_{\frac{{-w}^{'}}{2}+t^{'}}^{\frac{w^{'}}{2}-t^{'}} dx^{'}\int_{h^{'}}^{h^{'}+t^{'}} f^{2}\left( x^{'},z^{'} \right)dz^{'}+\int_{\frac{{-w}^{'}}{2}}^{\frac{{-w}^{'}}{2}+t^{'}} dx^{'}\int_{0}^{h^{'}+t^{'}} f^{2}\left( x^{'},z^{'} \right)dz^{'}+\int_{\frac{w^{'}}{2}-t^{'}}^{\frac{w^{'}}{2}} dx^{'}\int_{0}^{h^{'}+t^{'}} f^{2}\left( x^{'},z^{'} \right)dz^{'}}{\int_{{-x}_{0}^{'}}^{x_{0}^{'}} dx^{'}\int_{h^{'}}^{h^{'}+t^{'}} f^{2}\left( x^{'},z^{'} \right)dz^{'}}$ | (S8) |
| $F_{SA}^{'}=\frac{\int_{\frac{{-w}^{'}}{2}-2x_{0}}^{\frac{{-w}^{'}}{2}} dx^{'}\int_{-t^{'}}^{0} f^{2}\left( x^{'},z^{'} \right)dz^{'}+\int_{\frac{w^{'}}{2}}^{\frac{w^{'}}{2}+2x_{0}} dx^{'}\int_{-t^{'}}^{0} f^{2}\left( x^{'},z^{'} \right)dz^{'}}{\int_{{-x}_{0}^{'}}^{x_{0}^{'}} dx^{'}\int_{-t^{'}}^{0} f^{2}\left( x^{'},z^{'} \right)dz^{'}}$ | (S9) |

Figure S3d shows scaling factor of the MS interface as a function of $x_{0}^{'}$ position for different wiring cross-sections. The calculated participation ratios of the qubits used in this study are presented in Table S1.

**Table S1.** **Results of participation ratios for the transmon qubits used in the paper.**

| Qubit#  (etch/lift-off) | Design | $p_{{pads}_{MA}}$ | $p_{{pads}_{MS}}$ | $p_{{pads}_{SA}}$ | $p_{{leads}_{MA}}$ | $p_{{leads}_{MS}}$ | $p_{{leads}_{SA}}$ | $p_{{SQUID}_{MA}}$ | $p_{{SQUID}_{MS}}$ | $p_{{SQUID}_{SA}}$ |
| --- | --- | --- | --- | --- | --- | --- | --- | --- | --- | --- |
| 1/6 | long | 0.0362 | 0.5435 | 1.2723 | 0.1905 | 1.7733 | 1.3484 | 0.0353 | 0.3282 | 0.2496 |
| 3/4 | regular | 0.0487 | 0.7033 | 1.1862 | 0.0718 | 0.6679 | 0.5078 | 0.0399 | 0.3714 | 0.2824 |
| 5/2 | wide | 0.0170 | 0.4105 | 1.6588 | 0.0359 | 0.3605 | 0.2555 | 0.0398 | 0.4003 | 0.2837 |

Participation ratios values in the table are multiplied by ${10}^{4}$.

### II. Details on CHIP FABRICATION

The sample is fabricated on 525 $\mu m$ thick high-resistivity silicon. Firstly, the substrate is cleaned with a Piranha solution at 80 $℃$, followed by dipping in 2% hydrofluoric bath. Then 120 nm aluminum film is deposited using e-beam evaporation in an ultrahigh vacuum deposition system. After that, 600- $nm$ thick positive photoresist is spin coated. Then ground plane, resonators and qubit capacitors are defined using a laser direct-writing lithography system. Josephson junction wiring of the qubits of the «wet etched» group are also patterned in this step. Patterned features are then wet etched using commercial Al etchant solution. The photoresist is stripped in N-methyl2-pyrrolidone at 80 $℃$ for 3 hours and rinsed in IPA (isopropyl alcohol).

Josephson junctions, SQUID loops and wiring (for the «lift-off» group of the qubits) are then defined using Niemer-Dolan method. The process is described in details in Ref. [2,3]. The substrate is spin-coated with a resist bilayer composed of 500 nm MMA (methyl methacrylate) and 300 nm PMMA (poly methyl methacrylate). The development is performed in a bath of MIBK/IPA 1:3 solution followed by rinsing in IPA. Josephson junctions and wiring are patterned using an electron beam lithography system and then electrodes are shadow-evaporated in an ultra-high vacuum deposition system. First evaporated Al junction electrode is 25-nm thick and the second is 45-nm. Then aluminum bandages are defined and evaporated using the same process as for the junctions with an in-situ Ar ion milling. Lift-off is performed in a bath of N-methyl2-pyrrolidone with sonication at 80 $℃$ for 3 h and rinsed in a bath of IPA with sonication.

Finally, aluminum free-standing crossovers are fabricated for the suppression of parasitic modes, using a common fabrication process. 3 $\mu m$ photoresist is spincoated and then the sacrificial layer is patterned using a direct laser writing system. A 300 nm of Al is then evaporated with an in-situ Ar ion milling to remove the native oxide. The second layer of 3 $\mu m$ photoresist is used as a protective mask and the excess metal is wet etched. A damaged layer of photoresist is then removed in oxygen plasma and both layers of photoresist are stripped of N-methyl2-pyrrolidone at 80 $℃$.

### III. Details on EXPERIMENTAL SETUP

The detailed experimental setup scheme is shown in Fig. S4. The chip is connected to the control setup with seven lines: one line used both for readout and applying single qubit gates (XY controls) and six flux control lines used for detuning, coupled to each qubit. Pulsed XY control of the qubits was realized by upconverting the intermediate-frequency in-phase and quadrature signals from the arbitrary waveform generator (AWG), using IQ-mixer and microwave local oscillator. Detuning pulses were generated by single AWG channels. Readout tone was generated by AWG and up-converted to the readout resonator frequency using mixer and microwave local oscillator. The readout and XY control lines are combined by a 2-way splitter/divider. Readout microwave signal passes through the chip, is amplified by a cryogenic impedance matched parametric amplifier (IMPA) [4], and then downconverted. The readout signal is also amplified by high-electron mobility transistors (HEMT) at the 4K stage of the cryostat and at room temperature. We use a DC source and superconducting coil located on the packaging, to tune-up the parametric amplifier to the desired frequency and pump the IMPA by microwave source of vector network analyzer (VNA). Readout lines are additionally equipped with custom-made Eccosorb filters [5] on the cryostat mixing stage to suppress IR-noise and standing waves. Sample holders with qubit chip and IMPA are both placed in the magnetic shields.

| 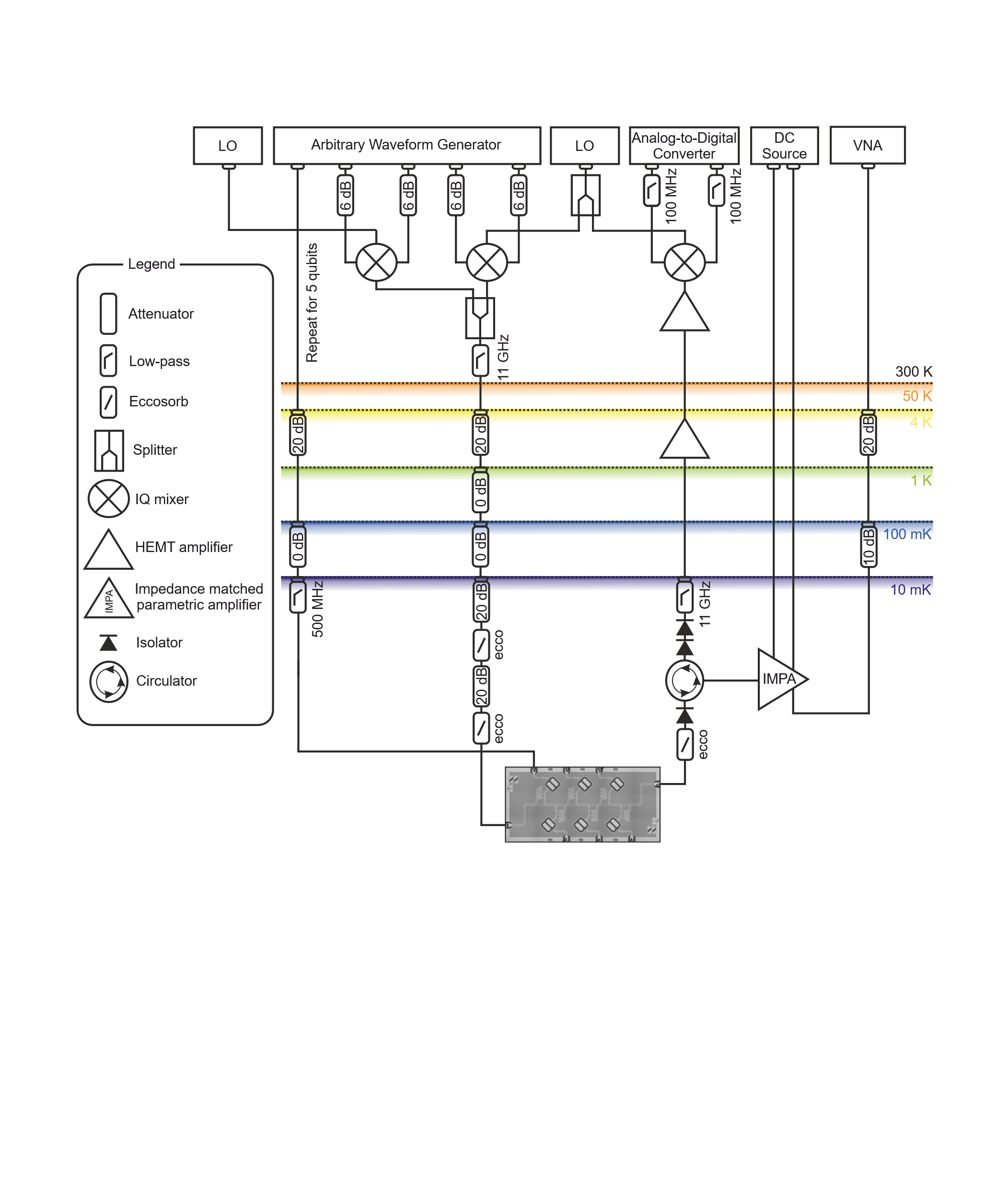 |
| --- |
| **Fig. S4 Schematic diagram of the experimental setup.** |

### IV. FITTING RELAXATION RESONANCES

### We identifed spurious lorentzian-like resonances on the measured Q-factor spectra of qubits Q1, Q3 and Q6. We fit qubit relaxation data to the Lorentzian function:

| $\frac{1}{T_{1}(f)}=A\frac{\Gamma}{\left( f-f_{0} \right)^{2}+\Gamma^{2}}+\Gamma_{Q}$ | (S10) |
| --- | --- |

### Lorentzian is parametrized by spurious resonance frequency$\boldsymbol{f}_{\boldsymbol{0}}$, scale $\boldsymbol{A}$, parameter specifying Lorentzian width $\boldsymbol{\Gamma}$ and qubit relaxaton rate $\boldsymbol{\Gamma}_{\boldsymbol{Q}}$ over the measurement range. We excluded the measured data points in a range of two Lorentzian peak widths $\boldsymbol{\Gamma}$ from the dielectric loss analysis, which we colored red in main text Fig. 3. All fitted parameters are consolidated in Table S2.

**Table S2.** **Fitted spurious resonances**

| Qubit | Data | A $\times{10}^{6}$ | $\Gamma$ (MHz) | $\Gamma_{Q}$ (MHz) |
| --- | --- | --- | --- | --- |
| Q1 | 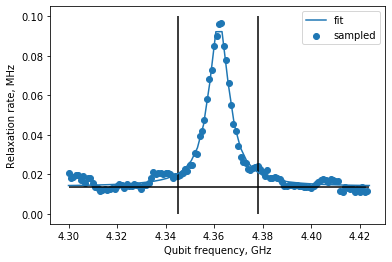 | 0.36 | 30.86 | 0.013 |
| Q3 | 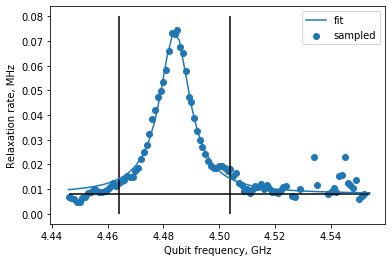 | 0.36 | 40.46 | 0.008 |
| Q6_1 | 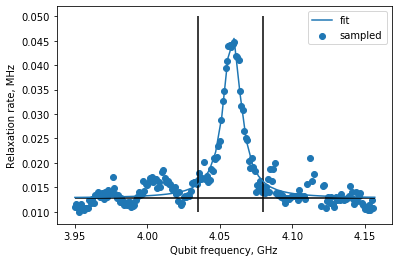 | 0.28 | 44.84 | 0.012 |
| Q6_2 | 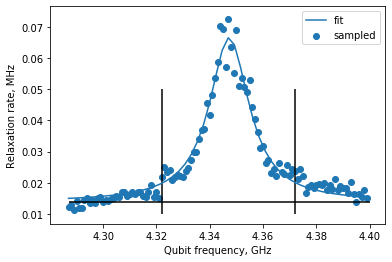 | 0.38 | 56,6 | 0.013 |

### V. Details on Monte Carlo simulation of qubit relaxation rate spectrum

To compare the experimental results with the TLS model, we performed a simulation of a qubit coupled to a TLS bath. Surface losses mainly arise from the interaction of qubits with a bath of incoherent TLS defects. The relaxation rate depends on the number of defects coupled to the qubit mode and its coupling strength. Here, we show that elements with even a relatively small footprint and, therefore, a small number of coupled TLS defects can significantly affect the quality factor of the qubit and have loss tangents close to those we obtained in this work. We simulate a qubit coupled with *i* TLS defects with the strength $g_{i}$, having relaxation rate $Г_{1TLS, i}$. We assume Markovian decoherence and calculate the qubit relaxation rate $Г_{1}$ [6,7]

| $Г_{1}= \sum_{i} \frac{2{g_{i}}^{2}Г_{1TLS, i}}{{Г_{1TLS, i}}^{2}+{\Delta_{i}}^{2}}$ | (S11) |
| --- | --- |

where $\Delta_{i}$ is detuning. The model assumes the limit $Г_{1TLS, i}>g_{i}$, where $Г_{1TLS, i}$is the defect relaxation rate. We simulate the distribution of electromagnetic field throughout the qubit using the same method as for calculating the participation ratios, applying the root mean square voltage to the qubit capacitive pads. We then sample random TLS defects from the dataset of Gaussian distributed dipole moments $d$ with mean dipole moment $d_{mean}=2.6 D$ and standard deviation $\sigma=1.6 D$ in accordance with the recent study [8]. We assign the decoherence rate to the defects, measured in Ref. [7,9–12], and a random detuning in the considered range of 300 MHz. We then place the sampled TLSs in the interface at a random position on the qubit to determine their coupling strength ($g=Ed$), and then using Eq. 10 calculate the spectrum of the qubit relaxation rate. To determine the amount of TLS, we took the previously obtained TLS density in amorphous alumina [13] $\rho_{o} \sim1800 {({\mu m}^{3}\cdot GHz)}^{-1}$ and set the effective thicknesses of the MA, MS, SA interfaces to 2, 0.3 and 0.36 nm, respectively. We assumed the MA interface thickness to be approximately equal to the native Al oxide interface thickness. The density of defects in the materials of the MS and SA interfaces is unknown, therefore the thicknesses of the MS and SA interfaces are set in accordance with the proportions of the interfaces loss tangents, experimentally extracted in Ref. [14] (${\tan\delta}_{MA}=3.9 \times{10}^{-3}, {\tan\delta}_{MS}=7.1 \times{10}^{-4}, {\tan\delta}_{SA}=5.9 \times{10}^{-4}$), since the loss tangent scales with the defect density [15] $\tan\delta=\pi\rho_{o}d^{2}{(3\epsilon)}^{-1}$. Note that the model according to Eq. S10 is used for strongly coupled defects, so we use it for the outer perimeter of capacitive pads and the ground plane, leads and SQUID, where the electric field is strong enough. For the inner areas of the pads with a weak electric field, we enter the background relaxation rate level, calculated using the Eq. 1 from the main paper with known loss tangents [16]. After simulating the relaxation rate spectra of qubits with three lead designs (“long leads”, “regular leads”, “wide leads”), we apply the SLE process as in the experiment and extract the loss tangents of the qubit elements: ${\tan\delta}_{pads}=(8.4\pm4.4)\times{10}^{-4}, {\tan\delta}_{leads}= (6.8\pm4.8)\times{10}^{-4}, {\tan\delta}_{SQUID}= (3.2\pm2.8)\times{10}^{-4}$. Simulated spectra and Q-factor histograms of the qubits converted from relaxation rates and rescaled to frequencies from 4 to 5 GHz are presented in Fig. S5a at 1 GHz bandwidth. The calculated quality factors from the extracted loss tangents vs. the simulated Q-factors are shown as a dashed line in Fig. S5b. Error bars represent 68% confidence interval. The shape of the predicted curve matches the simulated data and is similar to the curves obtained from the experiment. The loss tangents obtained from TLS simulation are similar to the experimentally extracted ones. The proportions between the loss tangents of the elements also correspond to experimental data. A comparison of the proposed model and experimental data indicates that even a small fraction of TLS’s located within the wiring interfaces of about 18% (total number of defects in the leads $N_{leads} \sim4300/GHz$ and in the SQUID loop $N_{SQUID}\sim950/GHz)$ have a significant contribution to total qubit dielectric loss and limit qubit relaxation time.

.

| 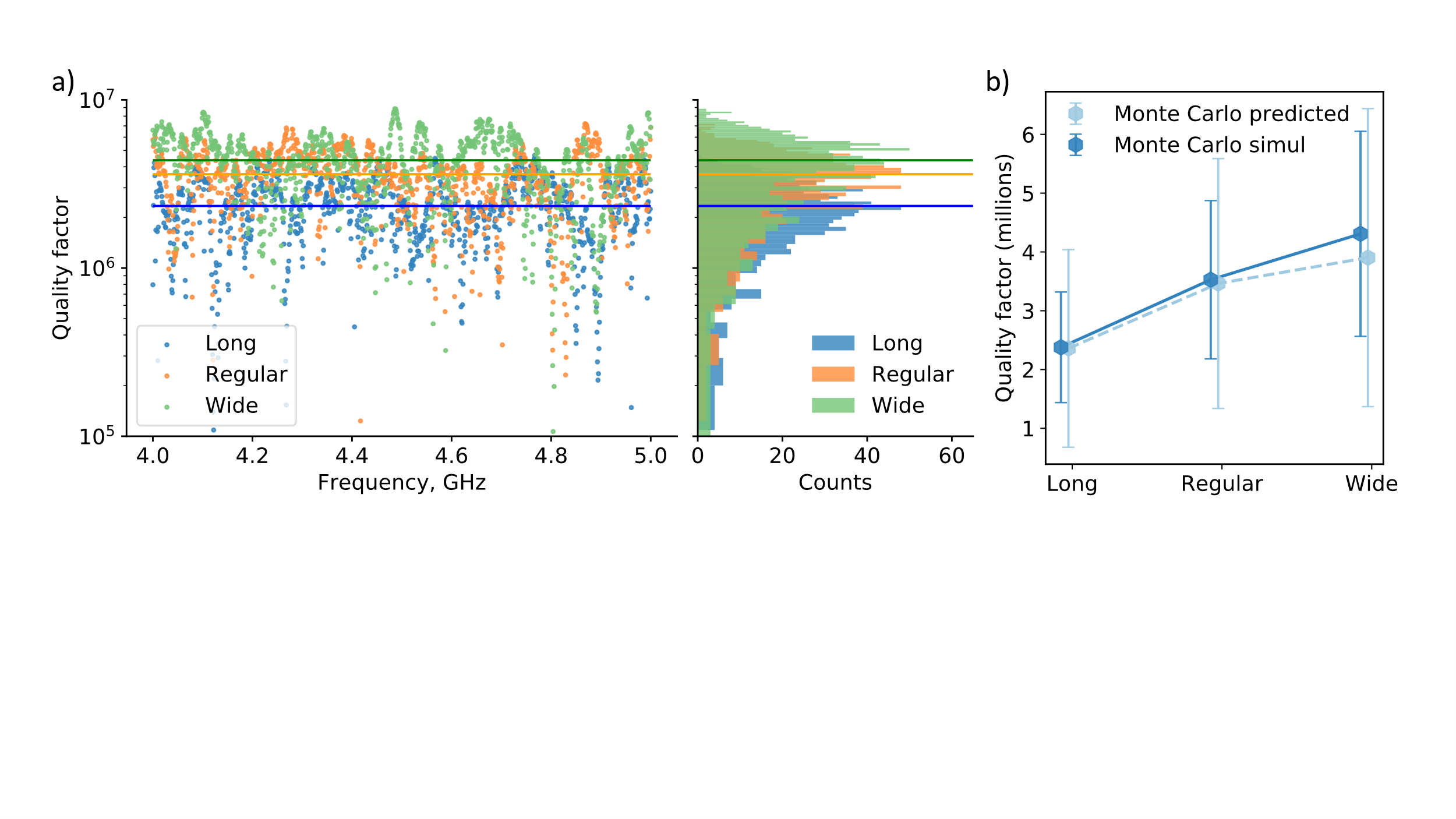 |
| --- |
| **Fig. S5 Monte Carlo simulation of defects in the qubits. a** Simulated Q-factor spectra and distribution. Blue, orange and green data correspond to the “long leads”, “regular leads” and “wide leads” respectively. Solid horizontal lines show the median Q-factor values of the corresponding qubit. **b** Monte Carlo simulated and predicted quality Q-factors as a function of leads geometry. Each data point represents median Q-factor obtained from the simulated qubit Q-factor spectra or sampled Q-factors using the SLE process. Error bars represent 68% confidence interval. Dark blue data points represent Q-factors from the simulated spectra. Light blue data points represent predicted Q-factors. |

**References**

[1] C. Wang, C. Axline, Y. Y. Gao, T. Brecht, Y. Chu, L. Frunzio, M. H. Devoret, and R. J. Schoelkopf, *Surface Participation and Dielectric Loss in Superconducting Qubits*, Appl Phys Lett **107**, 162601 (2015).

[2] D. O. Moskalev, E. V. Zikiy, A. A. Pishchimova, D. A. Ezenkova, N. S. Smirnov, A. I. Ivanov, N. D. Korshakov, and I. A. Rodionov, *Optimization of Shadow Evaporation and Oxidation for Reproducible Quantum Josephson Junction Circuits*, Sci Rep **13**, 4174 (2023).

[3] A. A. Pishchimova, N. S. Smirnov, D. A. Ezenkova, E. A. Krivko, E. V. Zikiy, D. O. Moskalev, A. I. Ivanov, N. D. Korshakov, and I. A. Rodionov, *Improving Josephson Junction Reproducibility for Superconducting Quantum Circuits: Junction Area Fluctuation*, Sci Rep **13**, 6772 (2023).

[4] D. Ezenkova et al., *Broadband SNAIL Parametric Amplifier with Microstrip Impedance Transformer*, Appl Phys Lett **121**, (2022).

[5] A. I. Ivanov, V. I. Polozov, V. V. Echeistov, A. A. Samoylov, E. I. Malevannaya, A. R. Matanin, N. S. Smirnov, and I. A. Rodionov, *Robust Cryogenic Matched Low-Pass Coaxial Filters for Quantum Computing Applications*, Appl Phys Lett **123**, (2023).

[6] R. Barends et al., *Coherent Josephson Qubit Suitable for Scalable Quantum Integrated Circuits*, Phys Rev Lett **111**, 080502 (2013).

[7] P. V. Klimov et al., *Fluctuations of Energy-Relaxation Times in Superconducting Qubits*, Phys Rev Lett **121**, 090502 (2018).

[8] C.-C. Hung, L. Yu, N. Foroozani, S. Fritz, D. Gerthsen, and K. D. Osborn, *Probing Hundreds of Individual Quantum Defects in Polycrystalline and Amorphous Alumina*, Phys Rev Appl **17**, 034025 (2022).

[9] J. Lisenfeld, C. Müller, J. H. Cole, P. Bushev, A. Lukashenko, A. Shnirman, and A. V. Ustinov, *Measuring the Temperature Dependence of Individual Two-Level Systems by Direct Coherent Control*, Phys Rev Lett **105**, 230504 (2010).

[10] M. Neeley, M. Ansmann, R. C. Bialczak, M. Hofheinz, N. Katz, E. Lucero, A. O’Connell, H. Wang, A. N. Cleland, and J. M. Martinis, *Process Tomography of Quantum Memory in a Josephson-Phase Qubit Coupled to a Two-Level State*, Nat Phys **4**, 523 (2008).

[11] Y. Shalibo, Y. Rofe, D. Shwa, F. Zeides, M. Neeley, J. M. Martinis, and N. Katz, *Lifetime and Coherence of Two-Level Defects in a Josephson Junction*, Phys Rev Lett **105**, 177001 (2010).

[12] G. Sun, Z. Zhou, B. Mao, X. Wen, P. Wu, and S. Han, *Entanglement Dynamics of a Superconducting Phase Qubit Coupled to a Two-Level System*, Phys Rev B **86**, 064502 (2012).

[13] A. Bilmes, S. Volosheniuk, J. D. Brehm, A. V. Ustinov, and J. Lisenfeld, *Quantum Sensors for Microscopic Tunneling Systems*, Npj Quantum Inf **7**, 27 (2021).

[14] G. Calusine et al., *Analysis and Mitigation of Interface Losses in Trenched Superconducting Coplanar Waveguide Resonators*, Appl Phys Lett **112**, (2018).

[15] Jiansong Gao, The Physics of Superconducting Microwave Resonators, 2008.

[16] A. Melville et al., *Comparison of Dielectric Loss in Titanium Nitride and Aluminum Superconducting Resonators*, Appl Phys Lett **117**, 124004 (2020).
